# Secretome Profiling of Atlantic Salmon Head Kidney Leukocytes Highlights the Role of Phagocytes in the Immune Response to Soluble β-Glucan

**DOI:** 10.3389/fimmu.2021.736964

**Published:** 2021-11-30

**Authors:** Dimitar B. Iliev, Guro Strandskog, Mehrdad Sobhkhez, Jack A. Bruun, Jorunn B. Jørgensen

**Affiliations:** ^1^ The Norwegian College of Fishery Science, UiT The Arctic University of Norway, Tromsø, Norway; ^2^ Department of Gene Regulation, Institute of Molecular Biology ‘Roumen Tsanev’, Bulgarian Academy of Sciences, Sofia, Bulgaria; ^3^ Department of Medical Biology, Proteomics Platform, UiT The Arctic University of Norway, Tromsø, Norway

**Keywords:** β‐Glucan, Atlantic salmon, leukocytes, mononuclear phagocytes, polymorphonuclear granulocytes, extracellular vesicles, exosomes

## Abstract

β‐Glucans (BG) are glucose polymers which are produced in bacteria and fungi but not in vertebrate organisms. Being recognized by phagocytic leukocytes including macrophages and neutrophils through receptors such as dectin-1 and Complement receptor 3 (CR3), the BG are perceived by the innate immune system of vertebrates as foreign substances known as Pathogen Associated Molecular Patterns (PAMPs). The yeast-derived BG has been recognized for its potent biological activity and it is used as an immunomodulator in human and veterinary medicine. The goal of the current study was to characterize the immunostimulatory activity of soluble yeast BG in primary cultures of Atlantic salmon (*Salmo salar*) head kidney leukocytes (HKLs) in which phagocytic cell types including neutrophils and mononuclear phagocytes predominate. The effect of BG on the secretome of HKL cultures, including secretion of extracellular vesicles (EVs) and soluble protein55s was characterized through western blotting and mass spectrometry. The results demonstrate that, along with upregulation of proinflammatory genes, BG induces secretion of ubiquitinated proteins (UbP), MHCII-containing EVs from professional antigen presenting cells as well as proteins derived from granules of polymorphonuclear granulocytes (PMN). Among the most abundant proteins identified in BG-induced EVs were beta-2 integrin subunits, including CD18 and CD11 homologs, which highlights the role of salmon granulocytes and mononuclear phagocytes in the response to soluble BG. Overall, the current work advances the knowledge about the immunostimulatory activity of yeast BG on the salmon immune system by shedding light on the effect of this PAMP on the secretome of salmon leukocytes.

## Introduction

β-glucans (BGs) are glucose polymers with 1,3; 1,4 or 1,6 β-glycosidic bonds produced by microorganisms and plants, such as yeast, mushrooms, bacteria, algae, barley and oat but not by vertebrate organisms (1). BGs have been recognized for their potent immunomodulatory properties and beneficial therapeutic potential in mammals (2) and fishes (3). In mammalian leukocytes, BGs activate complex intracellular signaling cascades leading to leukocyte activation and they also modulate the immune response to other stimuli (4). The best characterized mammalian receptors for BG are dectin-1 and complement receptor 3 (CR3, CD11b/CD18) (5); however other innate immune receptors, such as Toll-Like Receptor-2 (TLR2) have also been implicated in the response to BG (6).

The stimulatory activity of BG on the immune system includes augmentation of pathogen phagocytosis and killing through oxidative burst activity (7) as well as upregulation of immune mediators such as proinflammatory cytokines and chemokines (8, 9). The complex signaling networks activated by BG may lead to diverse responses depending on the BG molecular weight, branching and solubility as well as the particular receptors and cell types involved - reviewed in (10). For example, while in macrophages activation of dectin-1 by particulate BG induces a robust proinflammatory response (11), CR3-mediated activation by soluble BG may result in upregulation and secretion of TGF-β-laden extracellular vesicles (EVs) with anti-inflammatory properties (12). Adding to the intricacy of the BG-induced immune responses, it has been found that the BG-induced unconventional protein secretion, including EV release, is under the control of complex, inflammasome- and autophagy-dependent mechanisms (13).

Although neither an ortholog nor a functional analog of mammalian dectin-1 has been identified in lower vertebrates such as teleosts, studies on the effect of BG on the immune system of teleosts and mammals have indicated that the immune response to BG is conserved throughout the vertebrate evolution. In teleosts, the beneficial immune-regulatory properties of BG have been studied extensively showing that BG administration may result in resistance to a wide range of pathogens (14–16). *In vitro*, piscine leukocytes from different fish species respond to stimulation with BG by upregulation of reactive oxygen and nitrogen radicals, augmentation of neutrophil extracellular traps release and by induction of immune gene expression (17–20).

Previously, we have demonstrated that primary salmon leukocytes exposed to different agents, including eukaryotic DNA as well as TLR ligands such as CpG oligonucleotides (TLR9/21) (21) and R848 (TLR7/8), upregulate secretion of EVs containing diverse immune receptors and effector molecules (22). Mammalian EVs, in particular exosomes which are small (30-120nm) vesicles originating form endolysosomal compartments, serve as carriers of proteins, lipids and nucleic acids and are essential factors in communication between leukocytes and other cell types (23). In teleosts, studies on EV secreted by immune cells are still relatively scant.

The major aim of the current study is to characterize the secretome, including the protein content of small EVs and soluble proteins of primary Atlantic salmon HKLs stimulated with soluble yeast (*Saccharomyces cerevisiae*) BG by using Western blotting (WB) and high throughput mass spectrometry (LC-MS/MS). The data highlights the role of salmon phagocytes, such as neutrophils and mononuclear phagocytes, in the immune response to BG.

## Materials and Methods

### Fish

Atlantic salmon (*Salmo salar*) strain Aquagen standard (Aquagen, Kyrksæterøra, Norway) was obtained from the Tromsø Aquaculture Research Station (Tromsø, Norway). The fish were kept at about 10°C in tanks supplied with running filtered water and were fed on commercial, dry food (Skretting, Stavanger, Norway). All experiments were approved by the national committee for animal experimentation (Norwegian Animal Research Authority) and performed according to its guidelines.

### Reagents

Water-soluble yeast beta glucan was provided by Biotec Pharmacon (Tromso, Norway). Phosphorothioate-modified CpG class B (2006PS) oligodeoxynucleotides (ODNs) (5′-TCGTCGTTTTGTCGTTTTGTCGTT-3′) were purchased from Thermo Scientific. The salmon MHCIIβ antibody was produced in rabbit using a synthetic peptide: DGREVKSDVTSTEEL (22). Antibody against flotillin-1, was obtained from Abcam, Cambridge, UK (ab41927); actin was from Sigma Aldrich (A2066). Monoclonal antibody recognizing mono- and polyubiquitinated conjugates (P4D1) was purchased from Enzo Life Sciences, Lörrach, Germany (BML-PW0930). Secondary anti-rabbit (sc-2004) and anti-mouse IgG (sc-2005) HRP-conjugated antibodies were obtained from Santa Cruz Biotechnology, Santa Cruz, CA, USA.

### Cell Cultures and EV Isolation

Head kidney leukocytes were isolated as previously described (24). Briefly, the HK and the spleen tissues were passed through 100-μm pore size cell strainers (Falcon) in L-15 medium containing penicillin (10 U/ml), streptomycin (10 μg/ml), 2% fetal bovine serum (FBS), and heparin (20 U/ml). The resulting suspension was placed on a 25/54% discontinuous Percoll gradient and centrifuged at 400 × g for 40 min at 4°C. The cells at the interface were collected and washed twice in L-15 medium. The density of the leukocyte suspensions was adjusted to 7 × 10^6^ cells/ml and the cells were further incubated in 24-well plates in L-15, 5% FBS.

EVs were isolated through a centrifugation protocol (25). Briefly, conditioned supernatants were centrifuged sequentially at 500 g (10 min), 1500 g (15 min), 10 000 g (40 min) to remove cells, apoptotic bodies and smaller cell debris (22). The supernatants containing small EVs, such as exosomes were then filtered through 0.2 μm filters (VWR) and ultracentrifuged at 114 000 g for 2 h using a SW50.1 rotor and an Optima L-80 XP ultracentrifuge (Beckman Coulter, Krefeld, Germany). The pellets were resuspended in 5 ml PBS and centrifuged again at 114 000 g. After the washing, the pellets were resuspended in PBS and analyzed immediately or stored at −70°C until further use. The post-ultracentrifugation supernatants (SN) were concentrated with 3 kDa cutoff filters and the volume of the SNs was adjusted to the level in EV samples with PBS.

### Real-Time PCR Analysis

RNA from HKL cells was isolated using RNeasy Mini Kit (Qiagen). On-column DNase digestion was performed using RNase-Free DNase set (Qiagen). For each sample 100 ng of total RNA was reverse transcribed using the TaqMan Reverse Transcription Reagents kit (Applied Biosystems). The expression of *inf2* was analyzed with Power SYBR Green PCR Master Mix: the expression of *il1b*, *cd83* and *ef1ab* was detected with TaqMan Fast Universal PCR Master Mix (Applied Biosystems). The primer and the probe sequences are listed in **Table 1**. The reactions were run in duplicate and included 5 μl of fivefold diluted cDNA. The reaction protocol and the data analysis have been previously described (26). *ef1ab* expression was used as endogenous control and the data is presented as fold difference values as compared to the non-stimulated cells.

**Table 1:** Primer and probe sequences used for gene expression analysis.

	GB accession #	Forward primer
*tnf2*	ABG91800	Fwd: TGCTGGCAATGCAAAAGTA Rev: AGCCTGGCTGTAAACGAAGA
*cd83*	XM_014200893	Fwd: GTGGCGGCATTGCTGATATT Rev: CTTGTGGATACTTCTTACTCCTTTGCA Probe: CACCATCAGCTATGTCATCC
*il1b*	NM_001123582	Fwd: GCTGGAGAGTGCTGTGGAAGA Rev: TGCTTCCCTCCTGCTCGTAG Probe: TTGGAGTTGGAGTCGGCGCCC
*ef1ab*	XM_014141923	Fwd: TGCCCCTCCAGGATGTCTAC Rev: CACGGCCCACAGGTACTG Probe: AAATCGGCGGTATTGG

### Flow Cytometry

Control HKLs and cells incubated with CpG ODNs, BG and Brefeldin A for 24 h were resuspended in 500 μl of PBS at density of 2x10^6^ cells/ml and were stained for 10 min with 1 μM DRAQ5 (Cell Signaling Technology) to label all cells and 167 nM SYTOX Green stain (Invitrogen) to label dead cells with damaged plasma membrane. The samples were analyzed using FACSAria (Becton Dickinson) flow cytometer.

### SDS-PAGE, Silver Staining, and Western Blotting

EVs and cell pellets were lysed in LDS sample buffer (Invitrogen) supplemented with 50 mm dithiothreitol (DTT) and denatured at 70°C for 10 min. Samples derived from equal numbers of cells were run on NuPAGE Novex Bis-Tris 4–12% gels (Invitrogen) in MOPS running buffer. EV samples were stained with SilverQuest Silver Staining Kit (Invitrogen), according to manufacturer’s instructions. For WB, the proteins were transferred to PVDF membranes, blocked (Tris-buffered saline, 5% BSA, 0.1% Tween-20) for 1 h, and incubated overnight with primary Abs (1:1000 dilution) followed by 1 h of incubation with the secondary HRP-conjugated antibodies (1:10 000 dilution). The blots were developed with either SuperSignal West Pico or Femto Chemiluminesccent Substrates (Pierce, Rockford, IL, USA).

The densitometry was performed on selected exosome markers using the ImageJ software: (http://rsb.info.nih.gov/ij/index.html). Statistical analyses were performed using the GraphPad Prism 6 software (GraphPad Software, Inc., San Diego, CA, USA). The value of P < 0.05 was considered significant.

### Myeloperoxidase Activity Assay

The MPO activity in EV samples and post-ultracentrifugation supernatants was measured with MPO activity assay kit (Abcam, ab105136) following the standard manual. The absorbance at 412 nm was measured using a microplate reader (SpectraMAX Gemini EM, Molecular Devices).

### Proteomic Analysis

Protein samples were run on NuPAGE Novex Bis-Tris 4–12% gels for ~ 5 min. The gels were stained with Coomassie G-250 (Invitrogen) and the whole lanes (~ 1 cm long) containing proteins were cut and subjected to in gel reduction, alkylation, and tryptic digestion using 6 ng·μL^−1^ trypsin (V511A; Promega, Madison, WI, USA). OMIX C18 tips (Varian, Inc., Palo Alto, CA, USA) was used for sample cleanup and concentration. Peptide mixtures containing 0.1% formic acid were loaded onto a Thermo Fisher Scientific EASY-nLC1000 system and EASY-Spray column (C18, 2 μm, 100 Å, 50 cm × 50 μm). Peptides were fractionated using a 2–100% acetonitrile gradient in 0.1% formic acid over 50 min at a flow rate of 200 nL·min^−1^. The separated peptides were analyzed using a Thermo Scientific Q-Exactive mass spectrometer. Data were collected in data dependent mode using a Top10 method. The raw data were processed using the Proteome Discoverer 2.1 software (Thermo Scientific, Waltham, MA, USA). The fragmentation spectra were searched against a NCBI nr *S. salar* database downloaded 01/2017 using an in-house Mascot server (Matrix Science, London, UK). Peptide mass tolerances used in the search were 10 ppm, and fragment mass tolerance was 0.02 Da. Peptide ions were filtered using a false discovery rate set to 5% for protein identifications.

For the quantitation of the relative protein levels within the individual samples, the raw data were processed in the MaxQuant software v1.6.0.16 using label-free Intensity Based Absolute Quantification (iBAQ). Only proteins with minimum two identified peptides were included in the analysis. The relative iBAQ (riBAQ) values were calculated as the ratio of the individual protein iBAQ values vs. the sum of the iBAQ values of all of the identified proteins in the sample.

For the Gene Ontology (GO) analysis, the accession numbers of the identified proteins were mapped using the Retrieve/ID mapping tool of The Universal Protein Resource (UniProt) (www.uniprot.org).

## Results

### Upregulation of *tnf2*, *il1b* and *cd83* Expression by Soluble Yeast BG

**Figure 1:**
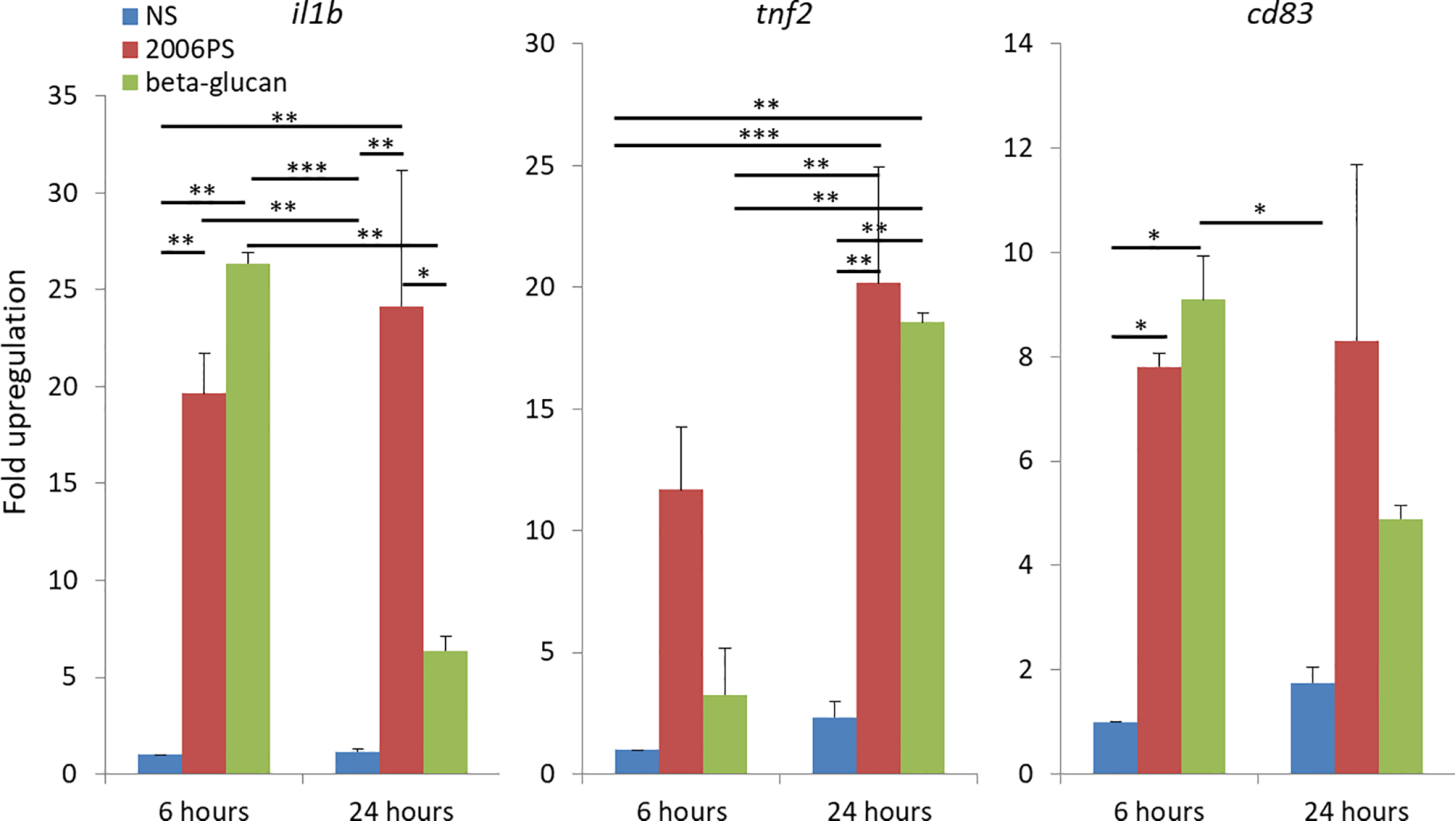
Soluble yeast BG induces a proinflammatory response comparable to that triggered by CpG ODNs as indicated by upregulated expression of immune genes in salmon HKLs. The cells were stimulated with 2 μM CpG and 50 μg/ml of soluble yeast BG for 6 and 24 h. The gene expression was analyzed with SYBR Green semi-quantitative Real-Time PCR. The values are presented as «fold upregulation» compared to the 6 h non-stimulated (NS) controls. The columns show mean values of three replicate experiments with cells from different fish; error bars - standard error. The data were analyzed with two-way ANOVA and Tuckey’s multiple comparison test; *P < 0.05, **P < 0.005, ***P < 0.0005.

In order to estimate the *in vitro* immunostimulatory capacity of soluble yeast BG, we did parallel treatments with BG and 2006PS CpG oligonucleotides (ODNs). This type of CpGs are potent inducers of immune gene expression in salmon HKLs (26, 27). We analyzed the expression of classical proinflammatory genes – *interleukin-1β* (*il1b*) and *tumor necrosis factor-2* (*tnf2*) as well as *cd83*. The latter is a marker for mature mammalian dendritic cells while in teleosts it plays a role in the induction of protective immune responses (24) and it is highly expressed in salmon APCs and activated granulocytes (26). The results presented in **Figure 1** show that treatment of HKLs with 50 µg/ml of BG induced a significant upregulation of all of the analyzed genes. The BG stimulation upregulated significantly *il1b* and *cd83* at 6 h and *tnf2* at 24 h of stimulation at levels comparable to those induced by CpGs.

### BG Induces Secretion of UbP Through a PI3K-Dependent Mechanism

**Figure 2:**
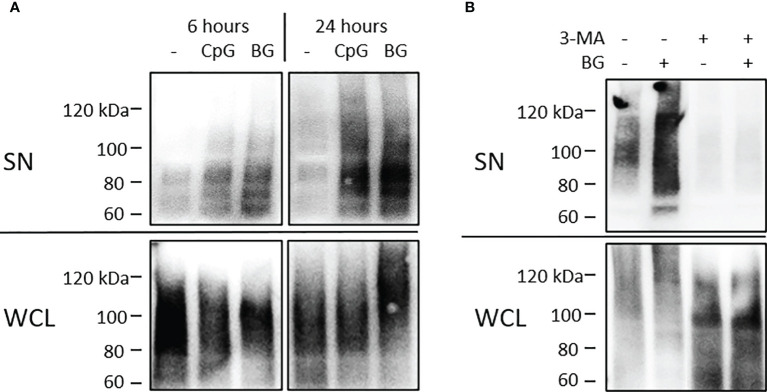
Soluble yeast BG induces secretion of UbP through a 3-methyladenine (3-MA)-sensitive mechanism. **(A)** HKLs were stimulated as in **Figure 1**. Supernatants and whole cell lysates (WCL) were precleared of cell debris and apoptotic bodies and analyzed on WB using anti-ubiquitin (P4D1) mouse mAb which detects free ubiquitin and ubiquitinated proteins. The UbP are visualized as a high MW smear (>60 kDa) in SNs (top) and whole cell lysates (WCL – bottom). **(B)** HKLs were stimulated with BG for 24 h with or without 10 mM 3-MA (a PI-3K inhibitor) and UbP levels in SN and WCL were analyzed as in **(A)**. Control cells were incubated with the same amount of vehicle (DMSO). The results shown in both panels were confirmed in at least 2 experiments with cells from different individuals.

UbPs are considered as markers for exosomes (28) and we have previously detected them in exosomes from HKLs stimulated with CpGs (27). The levels of extracellular UbP were analyzed in supernatants (SN) of HKLs stimulated as described in **Figure 1**. The conditioned SNs were precleared of cell debris and apoptotic bodies with sequential centrifugation at 500g and 1500g. The presence of UbP was detected with WB using an antibody which recognizes mono- and polyubiquitinated proteins. The results in **Figure 2A** demonstrate that, like CpGs, the BG-induced secretion of UbP is detectable after 6 h of stimulation and it is even more pronounced at 24 h of stimulation. As shown in **Figure 2B**, treatment of HKLs with 10 mM 3-metyladenine (a PI3K inhibitor) inhibited both the basal and the BG-induced secretion of UbP.

### Effect of BG on the Viability of HKLs

**Figure 3:**
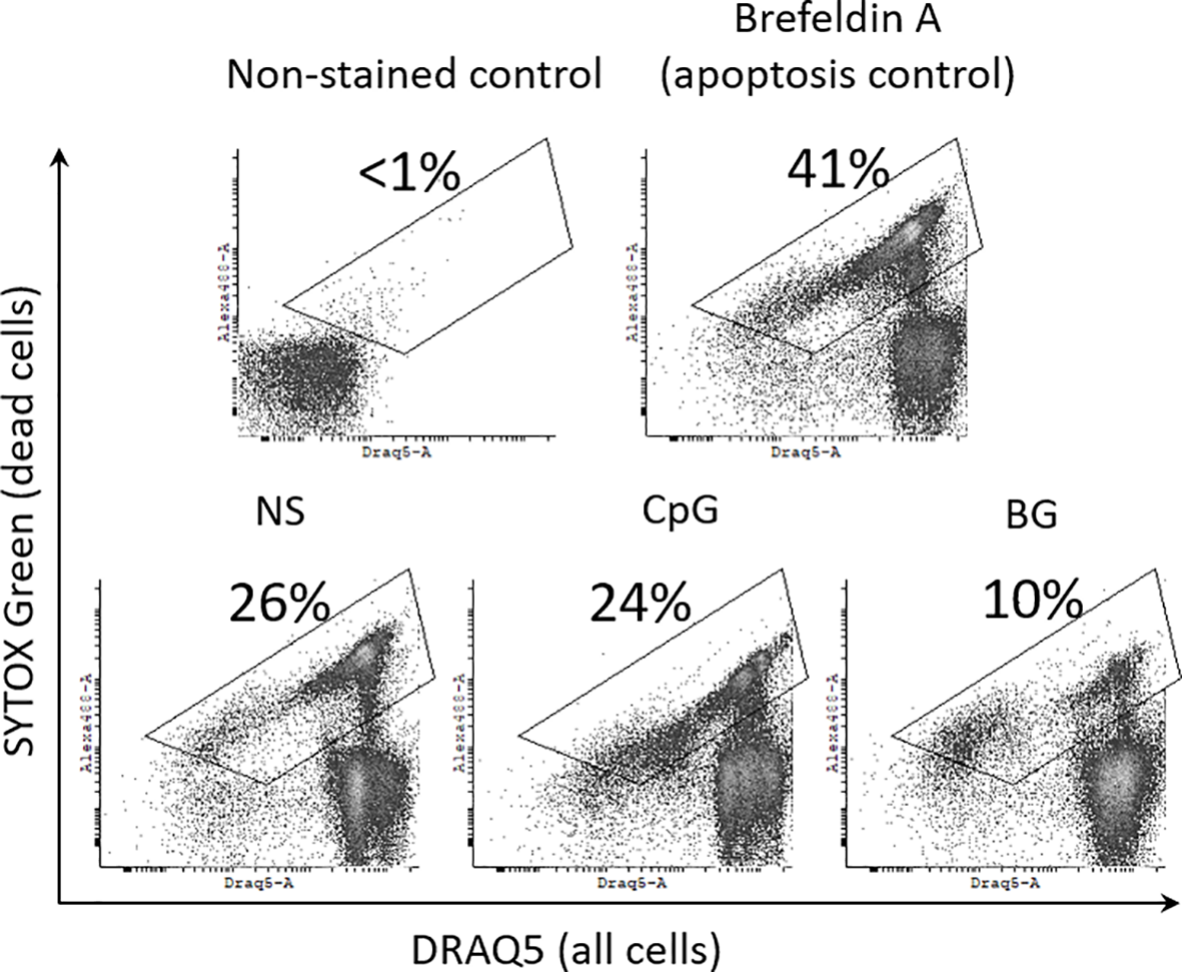
BG reduces the percentage dead cells and apoptotic bodies in HKL cultures. Cells were cultured for 24 h with 2 μM CpG, 50 μg/ml of BG and 10 mM Brefeldin A (a positive control for apoptosis). Dead cells with damaged plasma membrane, including necrotic and late apoptotic cells/apoptotic bodies were labelled with SYTOX Green, which does not stain live cells. The total cell population as well as the dead cells were stained with DRAQ5 which is a lipophilic, membrane permeable DNA stain. Non-stained control was used to set up the dead cell gate shown in the density plots. The percentage of dead cells and apoptotic bodies within the gate is displayed on each of the representative dot plots Similar results were obtained in two experiments with cells from different individuals.

In order to estimate the percentage of viable cells in HKL cultures we have used a differential DNA staining approach and flow-cytometry. SYTOX Green stains DNA only in dead cells including necrotic cells and late apoptotic cells with damaged plasma membrane. DRAQ5 is a lipophilic, membrane permeable DNA stain which labels both dead and viable cells. In the flow cytometry analysis, the dead cells, including necrotic cells and apoptotic cells/apoptotic bodies are double positive for SYTOX Green and DRAQ 5, while the viable cells are SYTOX Green-negative. The flow-cytometry data shown in **Figure 3** demonstrate that treatment of HKLs with 50 µg/ml of BG for 24 h leads to a decreased percentage of dead cells and apoptotic bodies (10%) as compared to non-stimulated HKLs (26%), while the CpG stimulation does not appear to affect the cell viability (24% dead cells). Treatment with 10 mM Brefeldin-A was used as a positive control for apoptosis (29), leading to an increased percentage of dead cells (41%).

### BG Induces Degranulation of PMNs in Primary HKL Cultures

**Figure 4:**
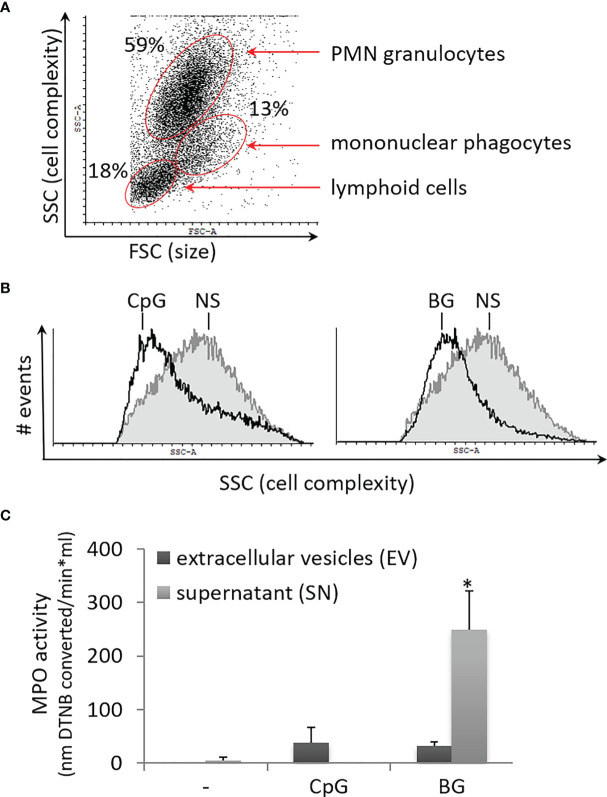
Soluble yeast BG and CpG ODNs induce degranulation of PMNs which is the predominant leukocyte type in primary HKL cultures. **(A)** The dot plot shows the setup of the gating of PMN granulocytes based on the side (SSC) and forward scatter (FSC) parameters. **(B)** The overlay histograms show the SSC values of PMN granulocytes gated as shown in **(A)** Non-stimulated control (NS) – filled grey contour; open black contour – cells stimulated as indicated for 24 h. Both BG and CpG stimulation decreased of the SSC values of the PMN population in three replicate experiments with cells from different individuals. **(C)** MPO activity is significantly upregulated in SN of HKLs stimulated for 24 h with 50 µg/ml of BG. The EV and SN samples were isolated as outlined in **Figure 5A**. The MPO activity was measured with a colorimetric assay based on conversion of DTNB. Error bars – standard error, n = 3. The data were analyzed with two-way ANOVA and Tuckey’s multiple comparison test; *P < 0.05, for all of the pairwise comparisons – i.e. the MPO activity was significantly upregulated in the supernatant of BG-stimulated cells compared to all other samples.

Previously, we have shown that salmon HKL cultures contain a high percentage of PMNs as well as mononuclear phagocytes with characteristic flow-cytometric forward (FSC) and side scatter (SSC) parameters in [(26), **Figure 4A**]. Upon stimulation with both BG and CpG ODNs, the cells in the PMN gate displayed reduced SSC values as compared to non-stimulated controls (**Figure 4B**). As previously observed in mouse granulocytes stimulated with PMA and in patients with COVID-19, reduction of the SSC values is indicative of neutrophil degranulation (30, 31). In the current study, the degranulation of BG-stimulated PMNs was also confirmed by an MPO activity assay. In supernatants from BG-stimulated cells, the MPO activity was significantly increased (**Figure 4C**). Higher MPO activity compared to control samples was also detected in EVs from CpG and BG-stimulated samples; however, the upregulation was not statistically significant.

### Soluble BG Induces Secretion of EV-Marker Proteins in Cultures of Salmon HKLs

**Figure 5:**
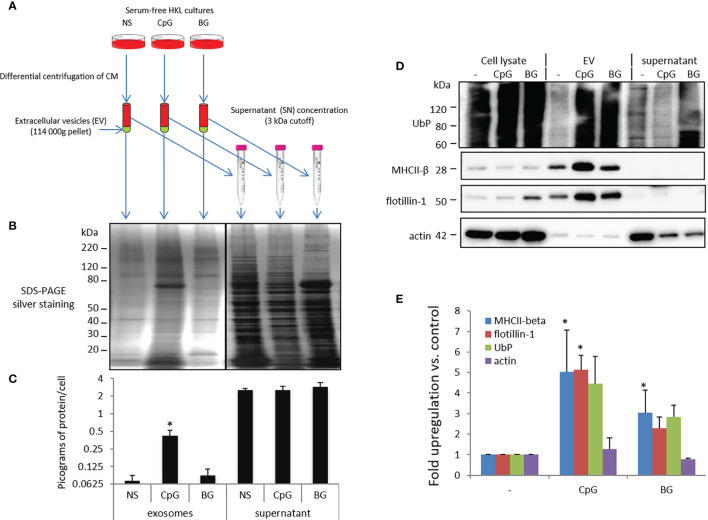
BG- and CpG-induced secretion of EV marker proteins. **(A)** Flowchart of the experimental setup. Conditioned supernatants of control non-stimulated cells (NS) and HKLs incubated with CpG ODNs and BG for 24 h in absence of FBS were subjected to differential centrifugation. After the ultracentrifugation step (114 000g for 2 h), the pellets were washed and resuspended in PBS. The post-ultracentrifugation supernatants (SN) were concentrated with 3 kDa cutoff filters and the volume of SN samples was adjusted to the level in EV samples with PBS. **(B)** Proteins from EVs and SN released from an equal number of cells were subjected to SDS-PAGE/silver staining. **(C)** The protein content of EVs and post-ultracentrifugation SNs was measured with a micro-BCA assay. **(D)** Proteins from EVs and SN released from equal numbers of cells as well as whole cell lysates (WCL) were subjected to Western blotting with the indicated antibodies. Representative results from three experiments with cells from different individuals are shown. **(E)** Western blot densitometry analysis. The intensity of the signal in the stimulated EV samples is presented as “fold upregulation” compared to the corresponding controls. N = 3, error bars – standard error. The data were analyzed with one-way ANOVA and Tuckey’s multiple comparison test; *P < 0.05 compared to non-stimulated control.

To investigate the effect of BG on secretion of EVs, samples were isolated as outlined in **Figure 5A**. Parallel stimulation with phosphorothioate CpG ODNs was included as a positive control for EV induction. The protein content of EVs and post-ultracentrifugation supernatants was analyzed with SDS PAGE/silver staining (**Figure 5B**) and micro-BCA (**Figure 5C**). Although BG induced a slight increase of the total protein content in ultracentrifugation pellets, the upregulation was far less pronounced as compared to that induced by CpG ODNs and was not statistically significant. The protein concentration in the post-ultracentrifugation supernatants was about an order of magnitude higher as compared to EVs and was not affected significantly by any of the stimulations.

The representative WB images shown in **Figure 5D** and the WB densitometry analysis (**Figure 5E**) show that the BG stimulation increased the amounts of the EV markers UbP, MHCII-β and flotillin-1 but not actin (a contaminant) in the ultracentrifugation pellets. The upregulation was statistically significant for MHCII-β in BG-stimulated samples and MHCII-β and flotillin-1 in CpG-treated samples when compared to the controls.

### Proteomic Analysis of EVs and Post-Ultracentrifugation Supernatants From Control and BG-Stimulated Samples

**Figure 6:**
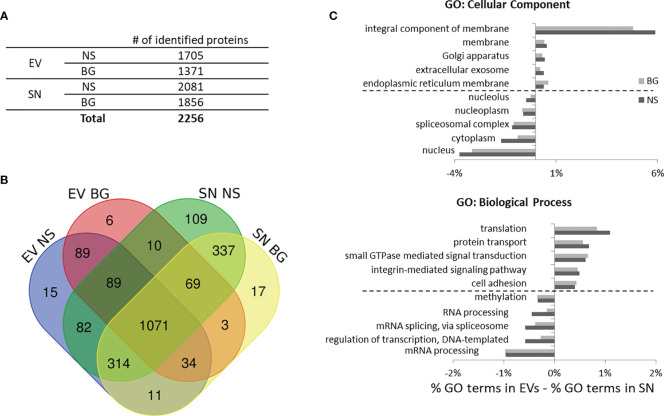
Identification of proteins in EV and SN samples from control non-stimulated (NS) and BG-stimulated HKLs through mass spectrometry. **(A)** Total number unique proteins identified in samples from two individuals. **(B)** Venn diagram showing the number of unique and common proteins identified in different samples. **(C)** Gene ontology (GO) analysis of the identified proteins in EVs and SNs from control and BG-stimulated cells. To identify features which are over- and under-represented in EVs vs. SNs, the percentages of the Cellular Component and Biological Process terms in the SN were subtracted from these in the EVs. The five top and bottom definitions over- and under-represented, respectively in EVs as compared to SNs are shown in the histograms.

To investigate the proteomic content of EVs and post-ultracentrifugation supernatants isolated as outlined in **Figure 5A** the samples were subjected to LC-MS with a Q-Exactive mass spectrometer as described in Materials and Methods. A total of 2256 unique proteins were identified in EV and SN samples from non-stimulated controls and BG-stimulated samples from two parallel experiments with cells from different individuals (the list with all of the identified proteins is presented in **Supplementary Table 1**. As seen in **Figure 6A**, higher numbers of proteins were identified in the SN as compared to EVs which reflects the total protein content in these samples (**Figure 5C**). Fewer proteins were identified in both EVs and SN of BG-stimulated samples as compared to controls. Most of the identified proteins (1683) were detected in both EVs and SN and only 110 proteins were discovered only in EVs vs. 463 found only in SN (**Figure 6B**). Despite the high number of common proteins, comparative Gene Ontology analysis (GO) (**Figure 6C**) demonstrated that there were substantial differences between the protein composition of EVs and SN. Within the “Cellular Component„ domain, the EVs were enriched in membrane-related classes and were depleted of “nucleus” and “cytoplasm” terms, as compared to SN. Within the “Biological Process” GO domain, “translation”, “protein transport”, “GTPase-mediated signaling”, “integrin-mediated signaling pathway” and “cell adhesion” terms were enriched in EVs while DNA and RNA-related classes were better represented in SN.

**Figure 7:**
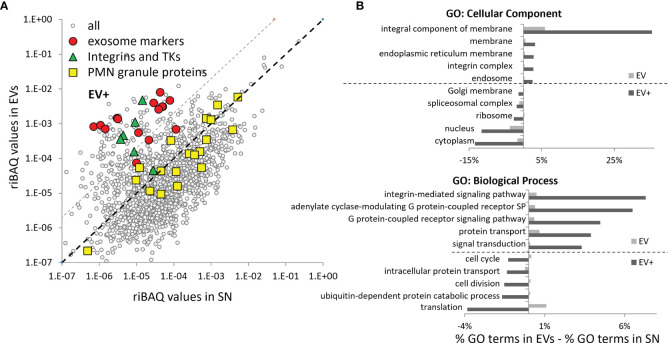
Comparison of the relative protein levels in EVs vs. SN – classical mammalian exosome markers and transmembrane proteins are enriched in EVs of salmon HKLs. **(A)** The average riBAQ values of the individual proteins identified in two samples of non-stimulated EVs were plotted against those in the SNs. Most of the classical EV markers, including some of the most specific exosome markers (according to exocarta.org), integrins and tyrosine kinases (TK) implicated in integrin signaling are enriched in the ultracentrifugation pellets. The highlighted proteins in each group are listed in **Figure 9**. Proteins which are more likely to be specific markers for salmon EVs and not contaminants were selected based on an arbitrarily set ≥20-fold higher riBAQ values threshold (grey dashed line) in EVs as compared to SN (EV+). **(B)** The GO analysis demonstrates that, in BG-stimulated samples, membrane-associated proteins and proteins involved in integrin-mediated signaling pathways are much more prevalent in the EV+ group as compared to the whole EV sample while nuclear and cytoplasmic contaminants underrepresented. The GO analysis is performed as described in **Figure 6**.

The label-free quantitation approach based on relative intensity-based absolute quantification (riBAQ) further highlighted the differences between the composition of EVs and SN (**Supplementary Table 2**). The riBAQ values of classical exosome markers as well as integrins and proteins involved in integrin signaling were much higher in EVs as compared SN (**Figure 7A**). GO Ontology analysis of a group of proteins with riBAQ values 20-fold ≥ in BG-induced EVs compared to SN (EV+) demonstrated enrichment of membrane and integrin-related classes and underrepresentation of nuclear and cytoplasmic contaminants compared to the whole EV sample (**Figure 7B**).

**Figure 8:**
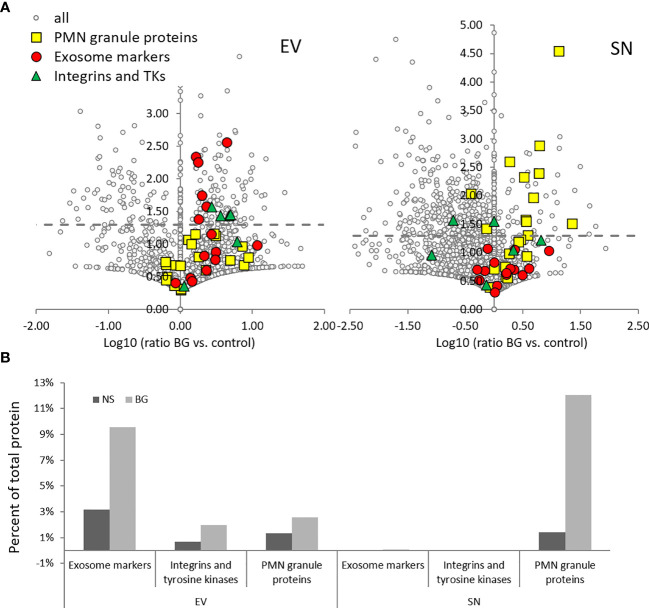
BG stimulation results in increased riBAQ values of exosome markers and integrins in EV samples and PMN granule proteins in SN as compared to non-stimulated (NS) controls. **(A)** Volcano plots showing the Log10 (ratio BG vs. control) on the x-axis, vs. –log10 (p value) on the y-axis of the riBAQ values in EVs (left) and SN (right). Grey dashed lines – p = 0.05 (Student’s t-Test); n = 2. **(B)** The histogram shows the sum of the riBAQ values in each of the highlighted groups shown in the volcano plots in **(A)**, presented as a percent of total protein content.

**Figure 9:**
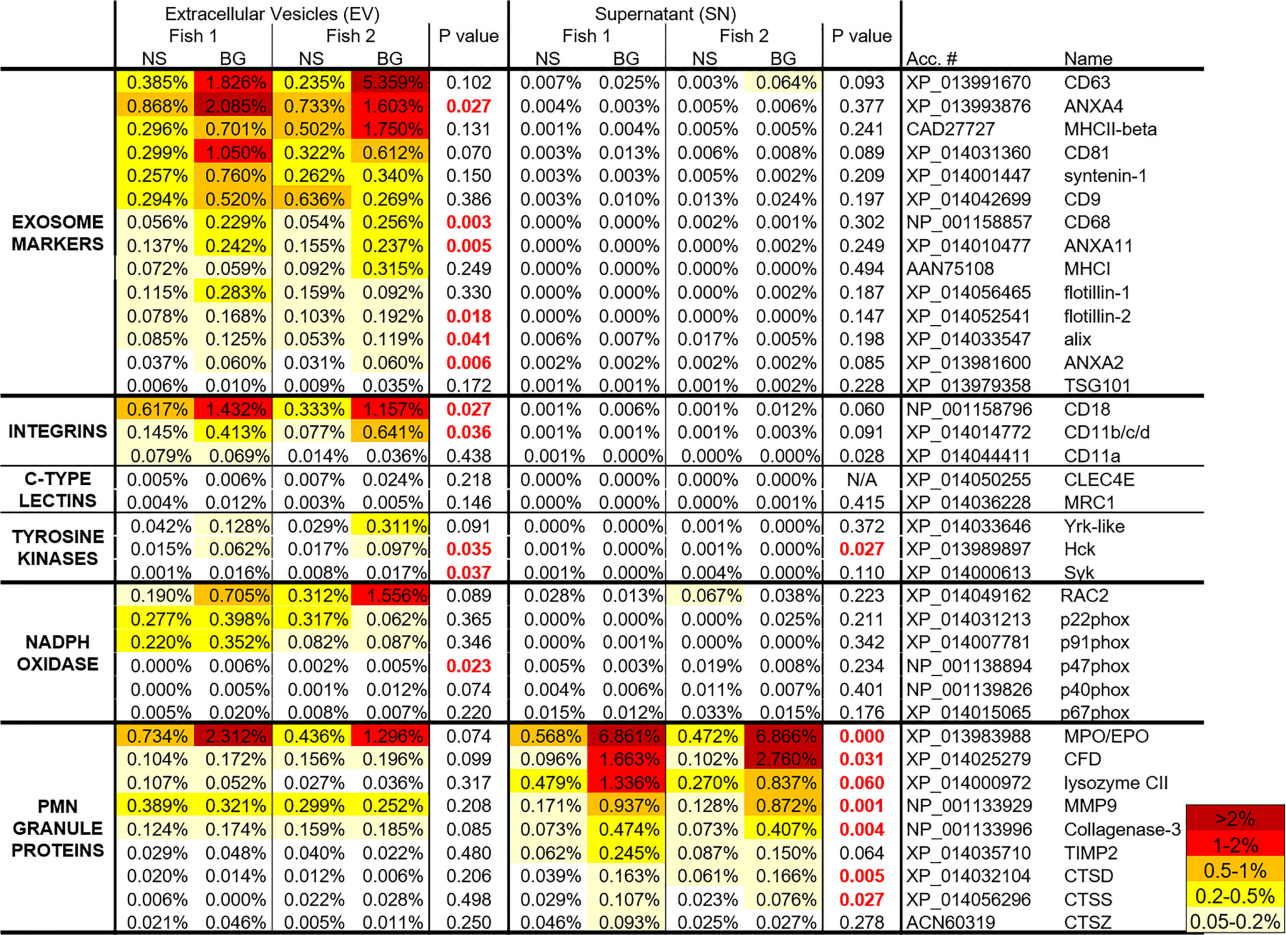
A heat map with the riBAQ values of the proteins in the highlighted groups in **Figures 7**, **8** as well as C-type lectins and NADPH oxidase components. The riBAQ values are presented as percentages of total protein content within the individual samples. The data was analyzed with Student’s t-Test. The p values < 0.05 are highlighted in red.

The effect of BG on the relative levels of individual proteins in EVs and SNs is shown in **Figure 8A**. In EVs, the stimulation upregulated the riBAQ values of selected exosome markers, integrins and tyrosine kinases involved in integrin signaling. Expectedly, in the SN of BG-stimulated cells, there was upregulation of PMN granule markers (**Figure 8B**). All of the highlighted proteins (except for PMN granule proteins with low riBAQ values) in **Figure 7** and **8** are listed in heat map shown in **Figure 9**. The list also includes NADPH oxidase components and C-type lectin homologs. Although the p values of most of the proteins of interest in EV samples were >0.05, the majority of them were consistently upregulated in the two replicate samples.

### Statistical Analysis

Data were analyzed with Student’s t-Test and one- or two-way ANOVA followed by Tuckey’s multiple comparison test as indicated in the figure legends.

## Discussion

In the current study, besides robust inflammatory gene upregulation, treatment of primary salmon HKLs with soluble BG induced considerable changes in the secretome of these cells. The latter was manifested by enhanced secretion of UbP, increased percentage of exosome markers as well as PMN granule proteins.

It has been suggested that UbPs may be regarded as markers for human and murine exosomes (28). In addition, in a previous study, we have demonstrated that UbPs are secreted within exosomes from salmon leukocytes stimulated with TLR ligands such as phosphorothioate CpG, R848 as well as control, non-immunostimulatory ODNs (22). While ubiquitination is important for targeting proteins for proteasomal degradation, it is also implicated in control of trafficking and sorting of membrane proteins as well as selective loading of specific proteins within EVs, including exosomes and plasma membrane-derived microvesicles (32). Remarkably, in HKL supernatants analyzed directly on WB, the levels of extracellular UbP were comparable between the CpG- and BG-stimulated samples whereas the upregulation of EV markers in ultracentrifugation pellets was considerably lower in the latter. In this regard, it is very likely that degranulating PMNs are the major source of extracellular UbP in the supernatants of BG-treated samples as it has been shown that UbP are abundant in primary PMN granules (33). Interestingly, although both CpGs and BG triggered PMN degranulation, the MPO activity was much higher in the supernatant of BG-stimulated samples, whereas in CpG samples it was detected mostly in EVs. While it is difficult to give a straightforward explanation of this difference, it is clear that the high BG-induced MPO activity in HKL supernatants reflects the potential of BG to induce antimicrobial defense mechanisms.

The BG-induced release of UbP by salmon HKLs is not due to passive leakage from dead cells since flow-cytometry analysis indicated that the BG treatment had reduced the percentage of dead leukocytes rather than increasing it. This could be explained by enhanced endocytosis of apoptotic cells by the phagocytes and/or the prolonged lifespan of the HKLs due to MAPK and NFkB signaling triggered by pattern recognition receptors (PRRs) (34). In further support, the BG treatment did not have a significant impact on the levels of the lactate dehydrogenase in cell culture supernatant (**Supplementary Table 2**) – indicating that the elevated concentration of UbP in cell culture medium is unlikely to be due to passive leakage from dead cells. Another indicator for enhanced viability of BG-stimulated HKLs, compared to control cells, is the relatively lower levels of extracellular cytochrome c, whose release is specific for apoptotic cells (35).

In mammals, the immune cells that respond to direct stimulation with BG are mostly phagocytic cell types including macrophages, neutrophils, monocytes and dendritic cells (36). In primary salmon HKL cultures, PMNs and mononuclear phagocytes are the most abundant cell types (26). Therefore, these cells are the major source of the proteins found in the secretome of the HKL cultures. Nevertheless, markers for lymphoid cells including CD22 and CD2 were also detectable in the EV samples (**Supplementary Table 1**) indicating that B- and T-lymphocytes may have also contributed to the pool of EVs.

Compared to CpGs, the stimulation with BG affected the EV secretion to a lower extent; nevertheless, the significant increase of the absolute levels of MHCII-beta in EVs from BG-treated samples indicates that the stimulation upregulated EV secretion from professional antigen presenting cells. This is further supported by the proteomic analysis showing increased relative concentration of classical exosome markers in BG-stimulated samples along with CD18 and CD11b/c/d homologs. The latter are particularly abundant in monocytes, macrophages and DCs (37). It should also be considered that, in exosomal preparations isolated through ultracentrifugation, considerable amounts of abundant intracellular proteins released from dead cells associate with and are co-purified at high levels along with the EVs. In this regard, the negative effect of the BG stimulation on the percentage of dead cells and apoptotic bodies (discussed above) might explain the lower number of proteins identified in supernatants and EVs from BG-treated samples. In contrast to contaminant proteins such as histones and actin, classical exosome markers had very high EV to SN riBAQ ratios. Many other proteins with similar ratio values have not previously been identified in exosomes and may, potentially, serve as specific markers for salmon EVs and exosomes, in particular (**Supplementary Table 2**).

It has been demonstrated that particulate BG induces respiratory burst activity in salmon macrophages (17). In addition, the NADPH complex is an important source for H_2_O_2_ necessary for MPO activity (38). In the current study, we have identified all of the major constituents of the neutrophil respiratory burst oxidase complex in the secretome of salmon HKLs. Unsurprisingly, in the control samples, the cytochrome b-245 alpha and beta chains (p22phox and p91phox, respectively), which form the constitutive membrane-associated element of the NADPH complex (NOX), were relatively more abundant in the EV fractions as compared to the supernatants while the opposite was observed for the cytoplasmic subunits of the complex - neutrophil cytosolic factor 1 (p47phox), 2 (p67phox) and 4 (p40phox). Upon activation, neutrophils translocate the cytoplasmic subunits, along with the GTPase RAC2 towards the plasmalemma and endosomal membranes resulting in the formation of the active NADPH oxidase complex (39). In the current study, the BG stimulation resulted in substantial upregulation of the relative amount of p47phox, p40phox and RAC2 within EVs which indicates that the soluble BG treatment has a potential to induce respiratory burst activity. These data also suggest that EVs from BG-activated salmon leukocytes might serve as carriers of active NOX and may play an important role in the respiratory burst activity against microorganisms. In addition, through production of ROS, NOX may participate in transcellular signaling events between leukocytes and other cell types. In this regard, it has been established that exosomes secreted by murine macrophages carry functional NOX which, upon entry in neurons, may initiate signaling events implicated in regulation of neuronal repair (40).

The inhibitory effect of 3-methyladenine (3-MA) on the BG-induced release of Ub-proteins indicates that this process is dependent PI3K signaling. Therefore, it is possible that the HKL response to BG might be initiated by receptors such as integrins and C-type lectins analogous to mammalian dectin-1 whose function depends on PI3K activity (41, 42). It has been confirmed that BG-specific receptors are present on teleost phagocytes as shown by the inhibitory effect of soluble BG on the phagocytosis of glucan particles, zymosan or whole yeast cells (43–45). So far *bona fide* orthologs of mammalian dectin-1 which, in mammals, is activated by insoluble but not soluble BG (46–48), have not been identified in teleosts (49). Another major mammalian receptor implicated in the recognition of BG is the complement receptor 3 (CR3) which is composed of a β2 subunit (CD18) and an αM subunit (CD11b) (50). The two subunits form a promiscuous receptor which, along with recognition of diverse extracellular matrix components as well as other endogenous ligands and PAMPs, is also involved in the immune response to soluble BG (51).

As mentioned above, we have identified homologues of the components of CR4 – namely, beta-2 integrin (CD18) and integrin-alpha X (CD11c) among the most abundant proteins present in the EVs which were further enriched in BG-induced EVs. In human, the functional properties of CR3 and CR4 are, by and large, overlapping; however, the carbohydrate-binding properties have, so far been ascribed to the alpha (CD11b) subunit of CR3 only. In this regard, it has been established that, during vertebrate evolution, the aM/aD/aX specialization has occurred after the divergence between teleosts and tetrapods (52). Although it remains to be determined, it is possible that the piscine homologs annotated as CD11c might bear the functional properties of the mammalian CD11b, including the BG-recognition. In addition to integrins, higher relative amounts of tyrosine kinases, including Syk, Hck and the particularly abundant Yes-related Yrk homologs were observed in the BG-stimulated EVs. Syk and Src tyrosine kinases are involved for the integrin-induced signaling (53) and preliminary experiments with a Syk inhibitor indicated that that the immunostimulatory activity of BG on salmon HKLs depends on Syk activity (**Supplementary Figure 1**). The data concurs with previously published results showing that particulate BG upregulates integrins and proteins involved in integrin signaling in EVs from human macrophages (54). This indicates that BG stimulation may intensify the transfer of active components involved in integrin signaling pathways to recipient cells.

In addition to induction of pathogen phagocytosis, respiratory burst activity and immune gene upregulation, the beta-2 integrins have been implicated in degranulation of PMNs (55). The CR3-dependent phagocytosis of yeast and PMN degranulation requires engagement of both the complement-binding I-domain and the carbohydrate-recognition lectin site of CR3 (56). Therefore, the PMN degranulation and upregulation of MPO activity in HKL supernatants might seem surprising as it would be expected to be induced by larger, opsonized BG particles but not soluble BG. In this regard, it has been found that shrimp C-type lectin (FcLec4a) acts as an opsonin to facilitate bacterial phagocytosis through interaction with beta-integrin (57). In the current study, salmon CLEC4E (Mincle) - a multifunctional soluble lectin (58) and mannose receptor C-type 1 (MRC1) homologs which are proteins with putative BG-interaction motifs (49) were enriched in EVs as compared to supernatants. It is possible that the response of salmon HKLs to BG might have been mediated by complex interactions of BG with multiple PRRs such as the above-mentioned C-type lectins and integrins.

In conclusion, the current study demonstrates that soluble yeast BG activates salmon leukocytes leading to degranulation of PMNs and secretion of UbPs and EVs from antigen-presenting cells. It should be acknowledged that a limitation of the current study is the lack of data from dose-response and time-course experiments. Nevertheless, the data obtained in the current study, in particular the large proteomic dataset, provides valuable information for the design of future trials aimed at addressing more specific questions about the biological activity of BG on salmon immune system.

## Data Availability Statement

The raw data supporting the conclusions of this article will be made available by the authors, without undue reservation.

## Ethics Statement

The animal study was reviewed and approved by the Norwegian Animal Research Authority.

## Author Contributions

Conceptualization: DI. Methodology, experimental design, and data acquisition: DI, GS, MS, JB, and JJ. Data analysis: DI and JB. Writing - original draft: DI. Writing - review and editing: DI, JJ, GS, and MS. Project administration: DI. All authors contributed to the article and approved the submitted version.

## Funding

The project was funded by the Research Council of Norway (Project No.: 230735/F20). DB received financial support from the Bulgarian National Science Fund (project No: КП-06-Н21/17–19.12.2018). MS received financial support from Biotec Pharmacon (Tromso, Norway). The funder was not involved in the study design, collection, analysis, interpretation of data, the writing of this article or the decision to submit it for publication.

## Conflict of Interest

The authors declare that the research was conducted in the absence of any commercial or financial relationships that could be construed as a potential conflict of interest.

## Publisher’s Note

All claims expressed in this article are solely those of the authors and do not necessarily represent those of their affiliated organizations, or those of the publisher, the editors and the reviewers. Any product that may be evaluated in this article, or claim that may be made by its manufacturer, is not guaranteed or endorsed by the publisher.
