# Supplementary material for: Secretome Profiling of Atlantic Salmon Head Kidney Leukocytes Highlights the Role of Phagocytes in the Immune Response to Soluble β-Glucan
Source: Front Immunol. 2021 Nov 30;12:736964. doi: 10.3389/fimmu.2021.736964 (PMC8671040; doi:10.3389/fimmu.2021.736964)
Supplement: Supplementary file 1 [file DataSheet_1.pdf]

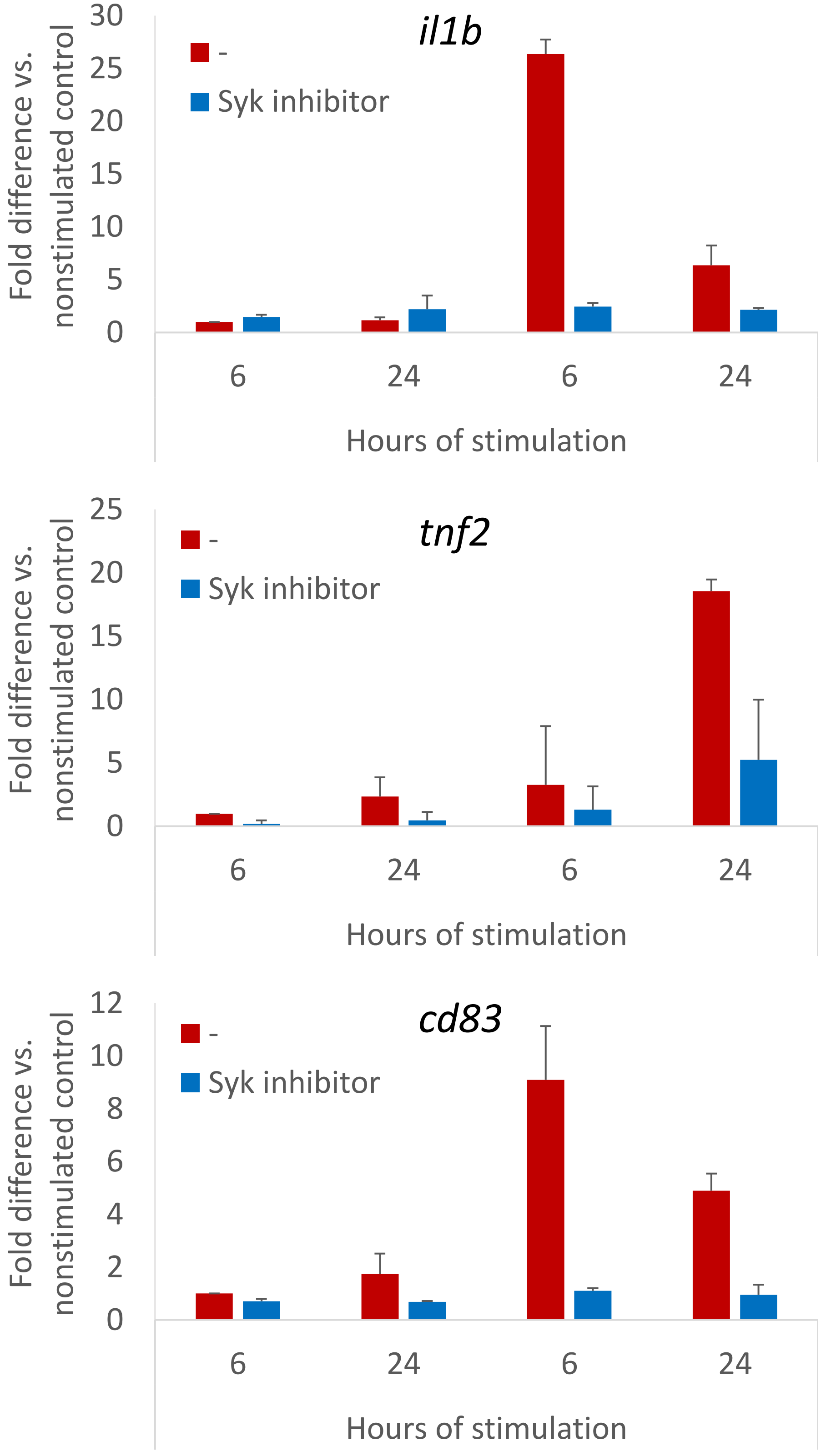

Supplementary figure 1. Syk inhibitor suppresses the BG-induced upregulation of *il1b*, *tnf2* and *cd83*. Prior to stimulation, the cells were pretreated for 30 min with 2 µM Syk inhibitor II (CAS 227449-73-2; Calbiochem) or an equivalent volume of vehicle (DMSO). The cells were then stimulated in the presence of inhibitor or vehicle and gene expression was analyzed as in Fig. 1. N=2, error bars - standard deviation.

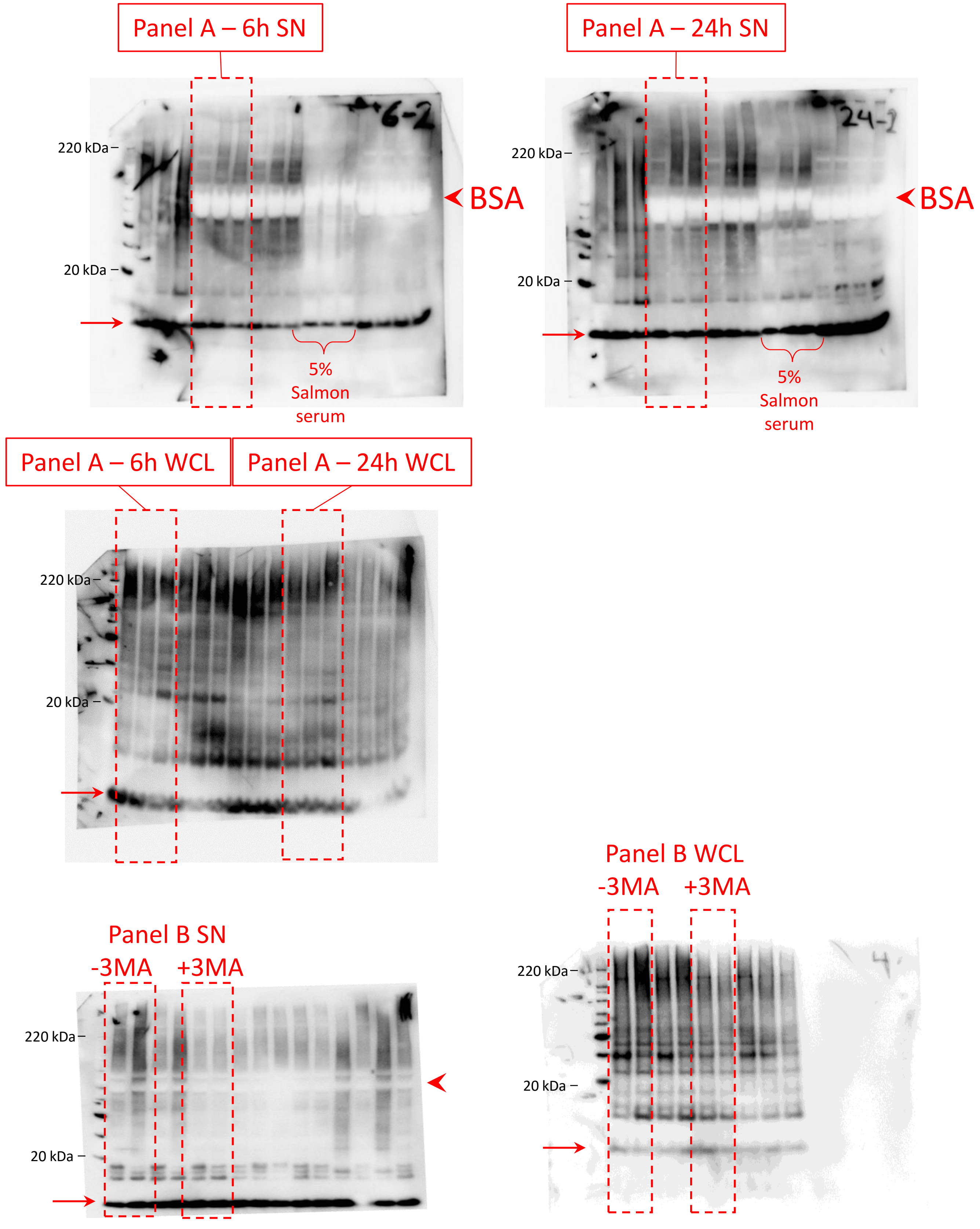

Supplementary figure 2., Unedited images of the WB results shown in fig. 3. The positions of the lightest and the heaviest Magic Mark XP bands are indicated on the left. The positions of the monoubiquitin bands (~7 kDa) are indicated with arrows. On the blots with supernatants containing FBS, the position of the BSA is visible as brighter spots (arrowheads) which confirm the equal loading of these samples. The blots containing the WCL samples were stained with Ponceau S as a loading control (not shown).
